# Usefulness of machine learning softwares to screen titles of systematic reviews: a methodological study

**DOI:** 10.1186/s13643-023-02231-3

**Published:** 2023-04-15

**Authors:** Ana Helena Salles dos Reis, Ana Luiza Miranda de Oliveira, Carolina Fritsch, James Zouch, Paulo Ferreira, Janaine Cunha Polese

**Affiliations:** 1 Post-Graduate Program of Health Sciences, Faculdade Ciências Médicas de Minas Gerais, Belo Horizonte, Minas Gerais, Brazil; 2 Faculty of Health Sciences, The University of Sydney, Sydney, NSW Australia; 3 Faculty of Medicine and Health, School of Health Sciences, Sydney Musculoskeletal Health, The Kolling Institute, The University of Sydney, Sydney, NSW Australia

**Keywords:** Citation screening, Text mining, Machine learning, Software tools, User Experience

## Abstract

**Objective:**

To investigate the usefulness and performance metrics of three freely-available softwares (Rayyan®, Abstrackr® and Colandr®) for title screening in systematic reviews.

**Study design and setting:**

In this methodological study, the usefulness of softwares to screen titles in systematic reviews was investigated by the comparison between the number of titles identified by software-assisted screening and those by manual screening using a previously published systematic review. To test the performance metrics, sensitivity, specificity, false negative rate, proportion missed, workload and timing savings were calculated. A purposely built survey was used to evaluate the rater's experiences regarding the softwares’ performances.

**Results:**

Rayyan® was the most sensitive software and raters correctly identified 78% of the true positives. All three softwares were specific and raters correctly identified 99% of the true negatives. They also had similar values for precision, proportion missed, workload and timing savings. Rayyan®, Abstrackr® and Colandr® had 21%, 39% and 34% of false negatives rates, respectively. Rayyan presented the best performance (35/40) according to the raters.

**Conclusion:**

Rayyan®, Abstrackr® and Colandr® are useful tools and provided good metric performance results for systematic title screening. Rayyan® appears to be the best ranked on the quantitative and on the raters’ perspective evaluation. The most important finding of this study is that the use of software to screen titles does not remove any title that would meet the inclusion criteria for the final review, being valuable resources to facilitate the screening process.

## What is new?

- Key findings

There are multiple machine learning tools that reviewers can use to facilitate and accelerate the title screening process while maintaining the quality of systematic reviews. The machine learning algorithms use reviewers’ relevance labels, keywords and text mining to predict which of the titles are relevant for the study.

- What this adds to what is known?

This study reported on the usefulness, performance metrics, and researcher's experience of three different machine learning softwares to semi-automated title screening process for systematic reviews.

- What is the implication, what should change now?

Rayyan®, Abstrackr® and Colandr® are useful, sensitive, and specific softwares to screen titles in systematic reviews, and can be safely used for title screening to save the workload and time of researchers. Overall, Rayyan® provided the best scores in the objective evaluation and on the raters’ perspectives.

## Background

Evidence-based practice is the pillar of decision-making for health professionals and integrates professional theoretical-practical knowledge, individual patient preferences, and high quality scientific evidence available in the literature [10, 14]. However, the search for the best literature for implementation in clinical practice is a complex process [17], especially considering the increase of over 2000% in the number of published studies over the last 20 years [11]. Furthermore, thousands of articles are published every year worldwide on different subjects and standards of care with varied levels of quality [23].

Systematic reviews are studies of high methodological quality developed through rigorous research processes that provide a reliable and valid summary of the available evidence on a specific subject, such as health interventions [6]. Systematic reviews are considered a reliable source to evaluate the quality and efficacy of health interventions, and improving the speed of development of these reviews is key to support evidence-based practice [1, 18].

Recently, several tools have been made available to speed up and facilitate the systematic review process. These tools help to reduce the costs needed to develop systematic reviews by decreasing manual work and time commitment of researchers through machine learning and text mining [1, 5]. Machine learning and text mining are features that refer to how softwares can learn through experience and repetition [5], while searching for keywords previously established by the researcher. Some softwares have been used to accelerate the process of a systematic review through semi-automatic screening of titles [3, 13]. However, the measurement properties and performance metrics of these softwares are still unknown, therefore they must be evaluated to help researchers understand if the softwares is suitable for title screening in systematic reviews when compared to the manual screening and which of the softwares is the most appropriate for this matter [18]. Therefore, the aim of this study was to investigate the usefulness and performance metrics of three freely-available softwares (Rayyan®, Abstrackr® and Colandr®) for title screening of systematic reviews.

## Methods

### Design

This was a methodological study that aimed to investigate the usefulness and performance metrics of three softwares (Rayyan®, Abstrackr® and Colandr®) for the title screening process of systematic reviews.

### Procedures

**Fig. 1:**
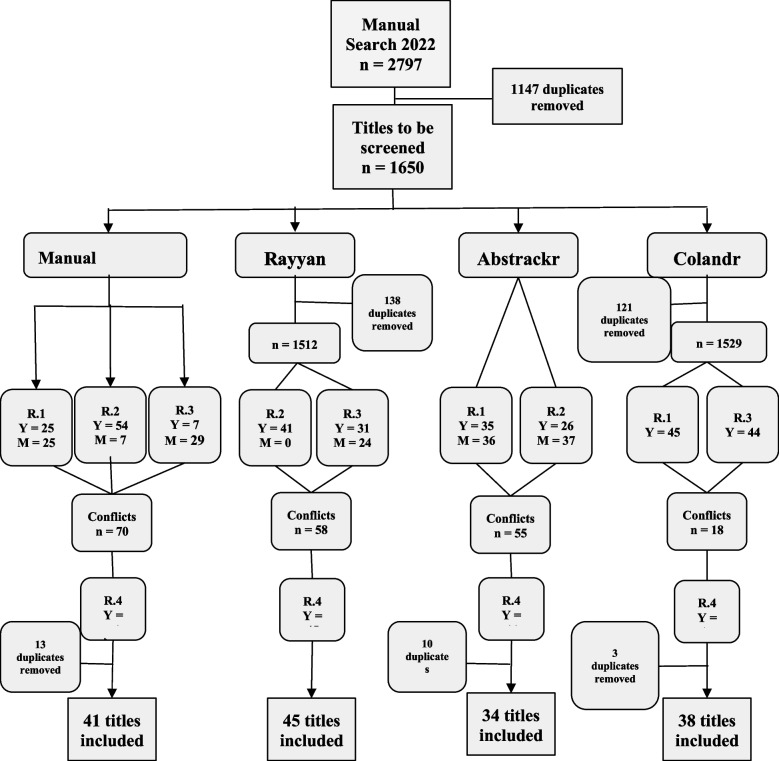
Flowchart of manual and software-assisted screening process. Description: Y = yes (titles that were included after the screening); M = maybe (titles that the rater had doubts whether to include or exclude); conflicts = when raters disagreed on the inclusion or exclusion titles; R.1 (AH); R.2 (CF); R.3 (AL); R.4 (JP)

To be included and evaluated in the current study, softwares had to fulfill the following criteria: (1) to be freely available; (2) to address health or multidisciplinary disciplines; (3) to use text mining or machine learning tools; and (4) to assist the process of screening of titles for systematic reviews. Subsequently, we used the study published by Harrison et al*.* [9] to support the selection of three highest-ranked softwares with the best scores on availability and screening features (Fig. 1). The three best-evaluated softwares, which were included in the current study, were: Rayyan®, Abstrackr® and Colandr®. Appendix 1 describes the characteristics of each selected software.

The screening process was initiated through manual screening and was followed by the software-assisted screening with an interval of three to seven days between screenings to avoid memory bias. The usefulness and performance metrics of the softwares were evaluated comparing the titles and the number of titles found on the manual screening and on the software-assisted screening. For the title screening process, manual screening was considered the ‘gold standard’ for comparison [12].

In the screening process, three raters, R.1 (AH), R.2 (CF) and R.3 (AL), with different experiences with the use of softwares and the systematic review process were chosen and allocated to use the softwares they had not had previous experience with. Each software was used by two different raters and a fourth rater (R.4, JP), who had the most expertise in the systematic review process, resolved all conflicts. After screenings, a short survey based on the Delphi technique [2] was developed to evaluate the rater’s subjective experiences regarding the softwares’ performances, as described in Appendix 2. The survey consisted of four questions concerning the process of learning how to use the softwares and their user-friendly and time-saving characteristics. Each question had three to five choice answers that ranged in scores from 0 to 10, with the final grade being the average of the two raters’ scores. This survey was pilot tested, updated and approved by 10 experts in the rehabilitation research field. The first three raters answered the questionnaire and their answers were analyzed by R.4, who summarized the scores of each software.

A systematic review in the musculoskeletal field, previously conducted and published by our research group [7], was chosen as the basis for the title screenings of the current study. The review investigated the effects of family-based interventions compared to individual-only interventions on pain intensity and disability of people with musculoskeletal pain. A total of 18 randomized controlled trials were included in the review following a manual screening process conducted by two independent researchers. The published search strategy is presented in Attachment A and was used to conduct the search strategy on Medline, Embase, PsycINFO, Amed, Web of science, PEDro and Cinahl databases. EndNote, a citation management software, was used to reduce the workload removing duplicates records using EndNote deduplication system and to download all records to each software via Research Information Systems’ files.

### Statistical analysis

The number of articles and the titles found through the manual screening and software-assisted screening processes were compared using a percentage agreement between them. As described by Valizadeh et al*.* [24], a performance metric assessment was conducted considering the values of true positives (TP), true negatives (TN), false positives (FP) and false negatives (FN) of each software. These results were manually calculated by the researchers. Regarding the included and excluded titles on each screening, the following formulas described by the same authors were used to calculate the metrics:

- Sensitivity = TP / (TP + FN).
- Specificity = TN / (TN + FN).
- Precision = titles correctly identified as relevant / all titles identified as relevant.
- False negative rate = titles incorrectly identified as irrelevant / all titles identified as relevant.
- Proportion missed = titles incorrectly predicted as irrelevant / all titles predicted as irrelevant.
- Workload savings = titles predicted as irrelevant / total titles to be screened.
- Time saving* = [(titles predicted as irrelevant × 0.5) / 60] / 8.

*As described by Valizadeh et al*.* [24], the estimated time saving is based on a screening rate of 0.5 min per title and an 8-h workday.

To describe raters’ subjective experiences with Rayyan®, Abstrackr® and Colandr® softwares, a descriptive analysis was used.

## Results

The current manual search process was carried out on the selected databases and identified 2797 titles, with 1650 remaining following the exclusion of the duplicates. As described on Fig. 1, all three raters firstly conducted a manual screening of titles on Excel and defined each title as included, excluded or maybe. Then, the fourth rater (R.4) resolved the conflicts and included 41 titles. Subsequently, two raters independently and blindly screened the titles on each software and each rater performed assisted screenings on two different softwares.

Raters R.2 and R.3 were responsible for the screening on Rayyan®, and each included 41 and 31 titles, respectively, and only R.3 selected maybe’s (*n* = 24). As Rayyan® detected the duplicates early on the process (*n* = 138), at the end of the screening, 58 titles were in conflict and 45 were included with no need to remove any more duplicates. R.1 and R.2 performed the screening on Abstrackr®, including 35 and 26 titles, each, with 36 and 37 maybe’s respectively. 55 titles were in conflict, and after R.4’s analysis and the late removal of 10 duplicates, 34 titles were included. Finally, R.1 and R.3 included 45 and 44 titles on Colandr®, with no maybe’s as the software does not allow this selection. It automatically removed 121 duplicates at the beginning of the screening, but after conflict resolution (*n* = 18), it required the late removal of three duplicates, totalling 38 included titles.

**Fig. 2:**
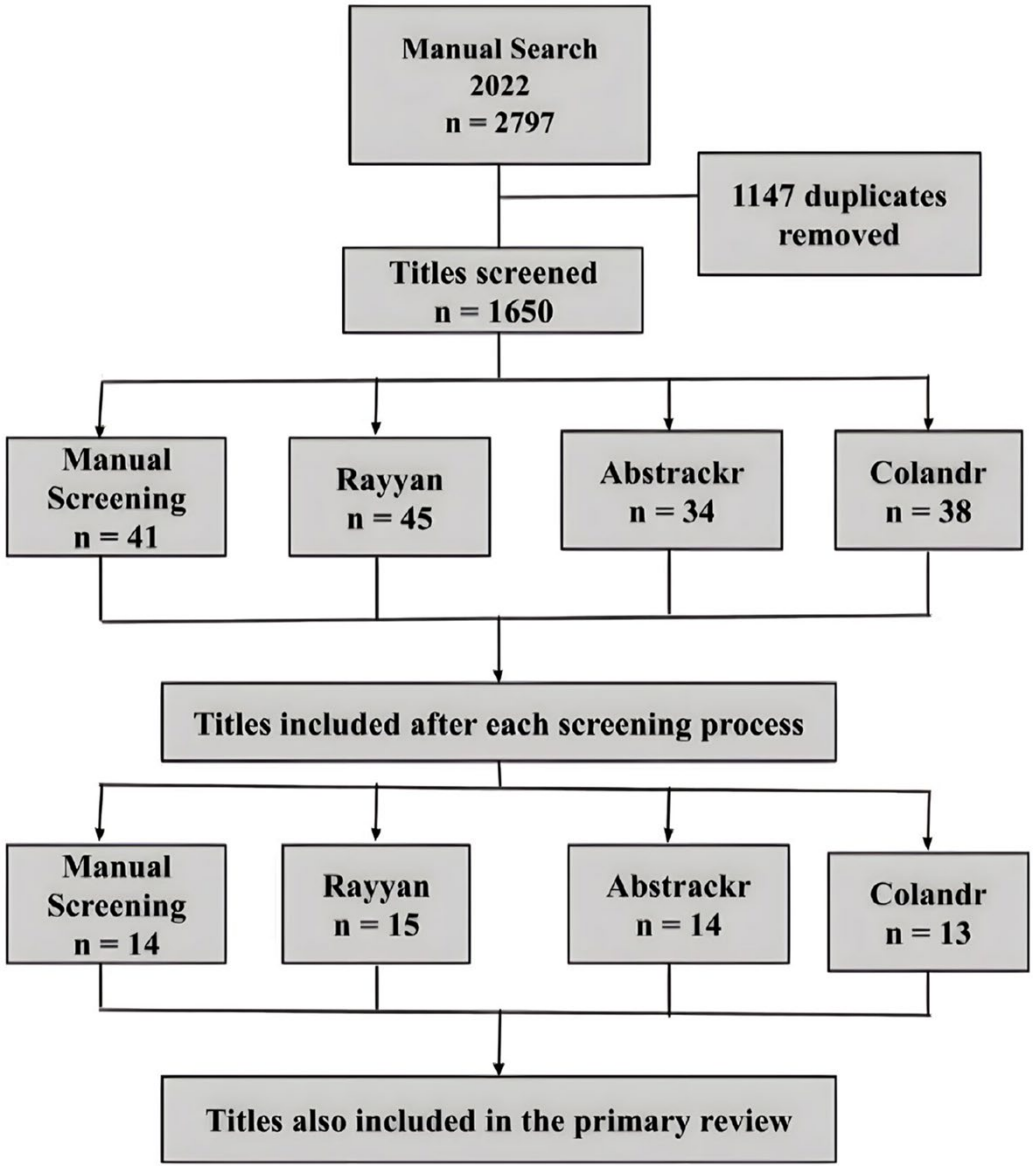
Flowchart of the comparison between the included titles on the first-level title screen and the final articles included in the review

In the primary review [7], researchers originally performed a manual search on the databases and found 1634 titles. After duplicates removal, 1223 titles were considered for title screening and eight extra citations were included after manual search. 18 titles were included in the review at the end of the screening process, being seven of them titles identified through manual search. Three out of the 18 titles were not found through the current databases searches and were therefore not included in the screening process. Thus, an average of 93.3% of the 15 titles included in both the primary review [7] and the current manual search were also included during the software-assisted title screenings on all three softwares (Fig. 2).

In the manual screening results, raters included 93.3% (14 titles) of the titles included in the primary review [7]. On Rayyan®, 100% were included (15 titles), while on Abstrackr® and Colandr® 93.3% (14 titles) and 86.6% (13 titles) were included, respectively, as described on Appendix 3.

Rayyan® was the most sensitive software, with the biggest proportion of titles correctly classified as relevant by raters (78%) using the software compared to those considered relevant on the gold standard method. Colandr® and Abstrackr® had sensitivity values of 65% and 60%, respectively. Rayyan® also had the lowest proportion of missed titles and false negative rate, scoring 0.5% and 21% respectively, while Colandr® scored 0.8% and 34%, and Abstrackr® scored 0.9% and 39%. All softwares had the same results for specificity, workload and time savings, indicating that semi-automated screening performed on the three softwares was faster than the manual screening (Table 1). Abstrackr® was the most precise software, followed by Rayyan® and Colandr®.

**Table 1:** Performance metrics of each software

Performance metric	Softwares		
	Rayyan	Abstrackr	Colandr
Sensitivity (SEN)	0.78	0.60	0.65
Specificity (SPE)	0.99	0.99	0.99
Precision	0.71	0.73	0.71
False negative rate (FNR)	0.21	0.39	0.34
Proportion missed	0.005	0.009	0.008
Workload saving	0.97	0.97	0.97
Time saving	1.67 days	1.68 days	1.67 days

After the screenings, all three raters answered the survey about the softwares performances (Table 2). The highest ranked software was Rayyan®, with a mean score of 35 out of 40 points, followed by Abstrackr® and Colandr® which scored 33.75 and 16.25 points, respectively. Even though it took over an hour for raters to learn how to use Colandr®, which was the least intuitive one, all softwares provided faster semi-automatic screening processes when compared to manual screening.

**Table 2:** Results of the survey on the softwares’ performances according to each researcher

QUESTIONS - Softwares performance	Rayyan		Abstrackr		Colandr	
	R.2	R.3	R.1	R.2	R.1	R.3
1- How would you rate the process of learning how to use the software?	10	7.5	7.5	10	2.5	2.5
2- In your experience, would you describe the software as user friendly (i.e. is it possible to understand the software instantly and intuitively)? On a scale of 0 to 10, where 0 is not friendly at all and 10 is very friendly, how would you rate it?	10	7.5	5	10	5	0
3- How long did it take you to learn how to use the software in minutes?	7.5	7.5	5	10	2.5	0
4- Was the semi-automatic screening process faster than the manual screening process?	10	10	10	10	10	10
**Total score:**	**35**		**33.75**		**16.25**	

## Discussion

Results from this study revealed that Rayyan®, Abstrackr® and Colandr® are useful tools for title screening in systematic reviews, although they all had different advantages and disadvantages. A very relevant result of this study is that the title screening softwares do not exclude any title that would meet the inclusion criteria for the final review, thus they are valuable resources to facilitate and accelerate the initial screening process. All softwares demonstrated adequate values of the investigated metrics, especially Rayyan®, which had the highest evaluation by raters. Rayyan® was the most sensitive software and raters were able to correctly identify 78% of titles deemed relevant on the manual screening, while Abstrackr® and Colandr® were sensitive to 60% and 65% of the titles, respectively. All softwares were specific, as raters correctly identified as irrelevant 99% of the titles deemed as irrelevant on the manual screening, and presented with precision of at least 71%. They incorrectly predicted as irrelevant less than 40% of the titles identified as relevant on the manual screening and were able to correctly predict as irrelevant 97% of all titles screened in all the softwares. Finally, the softwares were able to save 1.7 workdays, on average, “based on the citations that would not need to be screened” [4].

These findings mean that the softwares were able to predict and order the titles in terms of relevance, which sped up the screening processes since it was easier to include and exclude titles. This acceleration of the title screening process is necessary to keep up with the exponential growth of new publications [8]. Although all three softwares had good metrics values, relying exclusively on their predictions of relevancy may result in the oversight of relevant titles [22].

The differences between the raters regarding inclusion and exclusion of titles could be possibly explained by their different levels of experience with research. R.1 participated as research assistant in four systematic reviews processes, while R.2 had previously worked in seven systematic reviews and R.3 had no previous experience with title screening. However, this difference between raters’ inclusions and exclusions reduced in each screening as all of them learned the search strategies better throughout time. This was a strength of the current study as, despite their different levels of experience, they were still able to learn how to use the softwares.

Regarding the raters’ evaluations, Rayyan® was the most user friendly software, ranked in the performance questionnaire with 35 out of 40 points, agreeing with the findings of Ouzzani, et al*.* [19]. Raters took less time to learn how to use and navigate through the software’s interface, as well as to understand all of its features, as it is very intuitive. Rayyan® allows the user to choose terms for inclusion and exclusion, which might be highlighted on the titles to facilitate the screening, and it is very sensitive to detect duplicates [15, 19]. It also automatically updates the titles order by relevance every few inclusions and exclusions, as the machine learning process progresses. The software separates titles in different sections for the included, excluded, maybe’s and conflicts. One limitation identified was titles that were included after conflict resolution, remained on the conflict session and had to be counted separately. Conflicts could be seen by all raters only after blinding mode was off. Finally, Rayyan® was the only software which raters identified 100% of the studies that should be included on the screening, surpassing the gold standard, manual screening.

Abtrackr® was also highly rated in the questionnaire, ranked as the second best software, and raters could correctly identify 93.3% of the studies that should be included. Some of its positive features were that raters could be blinded, which is vital to reduce bias in the screening process. One researcher defined Abstrackr® as user friendly and praised the software for its good user interface with the best visibility of titles. It also did not demand a lot of time for the users to learn how to use it. Limitations identified included the absence of features to remove duplicate citations or highlights to favor text mining [4].

Colandr® was the least intuitive software and both raters required video guidance to learn how to navigate through its interface. In comparison with the other screenings, Colandr® was the least sensitive, although raters included 86.6% of the studies of the primary review. It was the only tool that required the definition of key terms and selection criteria for inclusion and exclusion of titles before the start of the screening process. Although this feature is positive as it allows the software to easily highlight these terms and to rank the titles by relevance, it also made rater’s work slower when compared to the other softwares, as it required a reason for each exclusion [9]. Also, users were not allowed to classify titles as “maybe”, which could reduce conflicts but might also be a reason for a larger number of incorrect inclusions and exclusions. Raters were not blinded, which increased the probability of bias as they were able to see all citations that were included, excluded and in conflict. Finally, Colandr® was sensitive to detect duplicate titles and removed them automatically, although a few had to be manually removed, making it a faster tool than the manual screening, similarly to the other softwares.

The findings of the current study show that these softwares can be used to facilitate the title screening process for systematic reviews, which are fundamental to the evolution of healthcare practices as they summarize the best available evidence [11]. In addition, the software proved to be a significant resource for not eliminating any title that should be included in the review, especially in title screening, which is usually the most tiring and time-consuming part of the process.

However, researchers must take into account that as softwares are managed by humans, they are also susceptible to errors, such as the omission of relevant titles or the inclusion of irrelevant ones [8]. Moreover, as Rayyan® and Colandr® were both able to detect duplicate citations, it would be feasible to upload all titles directly on the softwares, with no need of a citation management software such as EndNote.

### Limitations of the study

As only three softwares that met our inclusion criteria were evaluated, these findings should not be generalized to other softwares and abstract screening approaches. In addition, the results could be different with studies in a different field, with a higher number of citations to be screened or if the screening process was conducted by researchers with similar levels of experience with softwares and the systematic review process.

Another limitation is that the search strategy of only one systematic review was used to evaluate the softwares’ performance metrics. Using any of the softwares for the title screening process for different search strategies of different reviews could also influence these results.

Regarding the researchers' subjective experience of the softwares’ performances, the results cannot be generalized, since only three evaluators answered the questionnaire; and that the questionnaire contained only four questions on the topic. The differences between the TP and TN might be explained by human errors or biases, being unrelated to the softwares’ performances. Furthermore, as the softwares are regularly updated, there may be some changes to these findings if future studies are conducted, and they could test the softwares using different reviews to increase credibility.

## Conclusion

Rayyan®, Abstrackr® and Colandr® softwares are useful tools for title screening in systematic reviews and they all demonstrated adequate metrics values. Rayyan® appears to be the most sensitive software as it facilitated the identification of true positives by the raters and presented the least proportion of missed titles. When considering the practical use of the softwares, they had important differences regarding the subjective experience of raters. Rayyan® also had the highest score on the raters’ evaluation and was considered the most user friendly and intuitive of the three softwares, which is a relevant characteristic to be considered to increase researchers’ efficiency in conducting systematic reviews.

## Data Availability

All data generated or analyzed during this study are included in this manuscript.

## Appendix 1

**Table:** **Table 3** Characteristics of the softwares selected for the current study

*Software*	Description/site	Subject	Approach	Cost	Support	Score
*Abstrackr*	*Software* used of the title screening process of systematic reviews	Healthcare	*Text mining*	Completely free and free version available	Study selection Text analysis	Reorganizes the studies from 10% already screened
*Colandr*	Open access S*oftware* de acesso aberto to conduct the synthesis of evidence. It uses machine learning, natural language processing, and text mining functions to partially automate the location of relevant studies and extract the desired data from PDF articles	Multidiscipline	*Text mining* *Machine learning*	Completely free	Automatic search Study screening Data extraction	Every ten studies screened in the same review (including by multiple contributors), the software learns and the relevance ranking is updated
*Rayyan*	Collaborative web-based application to support conducting systematic reviews. It also includes a study screening mobile app	Multidiscipline	Visualization *Text mining* *Machine learning*	Completely free and free version available	Screening of studies Quality assessment Data extraction Collaboration Document management	*Rayyan* needs 50 screened titles with at least five included and five excluded to rank the relevance of the studies

## Appendix 2

Softwares performance questionnaire.

1. How would you rate the process of learning how to use the software?
  - Very easy (10 points)
  - Easy (7.5 points)
  - Not easy but also not difficult (5 points)
  - Difficult (2.5 points)
  - Very difficult (0 points)
2. In your experience, would you describe the software as user friendly (i.e. is it possible to understand the software instantly and intuitively)? On a scale of 0 to 10, where 0 is not friendly at all and 10 is very friendly, how would you rate it?
  - Very Friendly (10 points)
  - Above Average (7.5 points)
  - Average (5 points)
  - Below Average (2.5 points)
  - Not friendly at all (0 points)
3. How long did it take you to learn how to use the software in minutes?
  -  < 15 min (10 points)
  - 15 to 30 min (7.5 points)
  - 30 to 45 min (5 points)
  - 45 to 60 min (2.5 points)
  - > 60 min (0 points)
4. Was the semi-automatic screening process faster than the manual screening process?
  - Yes, with both softwares. (10 points)
  - Yes, but with just one of the softwares. (5 points)
  - No, both screening processes were similar regarding the time. (0 points)

Maximal score: 40 points.

Minimal score: 0 points.

## Appendix 3

**Table:** **Table 4** Comparison of studies included in the primary review with those included in each screening process conducted in the current study

Studies included in the primary review	Included on Manual Screening	Included on Rayyan	Included on Abstrackr	Included on Colandr
Abbasi et al. 2012	Yes	Yes	Yes	Yes
Kole-Snijders et al. 1999	No	No	No	No
Saarijarvi et al. 1991	Yes	Yes	Yes	Yes
Saarijarvi et al. 1991	Yes	Yes	Yes	Yes
Saarijarvi et al. 1992	Yes	Yes	Yes	No
Turner et al. 1990	No	No	No	No
Buchanan et al. 2017	No	No	No	No
Keefe et al. 1996	Yes	Yes	Yes	Yes
Keefe et al. 1999	Yes	Yes	Yes	Yes
Keefe et al. 2004	Yes	Yes	Yes	Yes
Martire et al. 2008	Yes	Yes	Yes	Yes
Martire et al. 2003	Yes	Yes	Yes	Yes
Martire et al. 2007	Yes	Yes	Yes	Yes
Moore & Chaney 1985	Yes	Yes	Yes	Yes
Ramke et al. 2016	Yes	Yes	Yes	Yes
Radojevic et al. 1992	Yes	Yes	Yes	Yes
Riemsma et al. 2003	No	Yes	Yes	Yes
Lomholt et al. 2015	Yes	Yes	No	No

## Attachment A

Systematic review search strategy: Fritsch CG, Ferreira ML, Silva AKF, Simic M, Dunn K, Campbell P, Foster NE, Ferreira PH. Family-based Interventions Benefit Individuals With Musculoskeletal Pain in the Short-term but not in the Long-Term: A Systematic Review and Meta-Analysis. Pain. 2021 Fev; Volume 37, Number 2, 140—157.
